# Continuity of care: betrayed values or misplaced nostalgia

**DOI:** 10.5334/ijic.1056

**Published:** 2012-10-25

**Authors:** Martin Roland

**Affiliations:** Institute of Public Health, University of Cambridge, Forvie Site, Robinson Way, Cambridge CB2 0SR, UK

**Keywords:** continuity of care, integrated care, family practitioner

## Abstract

Care is better coordinated when doctors have personal responsibility for their patients. Continuity and a sense of personal responsibility are becoming more difficult to provide in hospitals, in part because of the European Working Time Directive. However, in many countries general practitioners are self-employed and able to organise their practices as they wish. In the UK, they increasingly do so in ways that make it difficult for patients to get continuity of care. This is despite most patients being clear that they want to see a regular doctor, and professional bodies in primary care consistently promoting continuity as a core value. General practitioners need to decide whether continuity of care matters. If it does, then they need to take a lead in ensuring that care is organised so that patients who want to see a regular doctor are able to do so. Suggestions are included for how contemporary practice can be organised to promote this traditional but still highly relevant value.

The UK had a television programme in the 1960s with a theme which is probably familiar to viewers in many countries. It showed the life of a country doctor, pillar of the community, loved by his patients, confidant of all their inner secrets and shrewd detective when it came to a biopsychosocial perspective on life (though of course it wasn’t called that). Dr Finlay was the model doctor.

In a recent *Perspectives* article on continuity of care, George Freeman outlined the potential benefits of continuity to patients [1]. He drew attention to some of the gaps in our knowledge and identified areas where more research was needed to identify the outcomes of good continuity of care. In this paper, I look at continuity of care from the doctor’s perspective. I argue that doctors are confused, continuing to aspire to something called ‘continuity of care’ but increasingly failing to enact it in the way they organise their working lives. They are unsure whether Dr Finlay represents the true values of generalism, or merely a naïve and misplaced nostalgia.

Continuity of care is conventionally described in terms of three concepts: relational continuity, informational continuity and management continuity [2]. The last two have become more important as care has become more complex and more fragmented. When patients had a single doctor who knew all their problems, informational and management continuity were provided by the doctor’s knowledge of the patient. When care becomes delivered by multiple providers, then systems to ensure informational and management continuity become critical to patient safety. The problems that this can produce have become most obvious in European hospitals where the European Working Time Directive has led to restrictions on doctors’ working hours and the inevitability of shift rotas, with doctors sometimes looking after large numbers of patients with whom they are unfamiliar. Indeed, 28% of physicians in England currently say that their hospital’s ability to provide continuity of care as ‘poor’ or ‘very poor’ [3]. This has led to repeated professional calls for the European Working Time Directive to be interpreted more flexibly, but not to a public outcry at what in the US, Burstein describes as ‘a critical frontier in patient safety’ [4].

General practitioners in the UK are self employed and hence not subject to European regulations in the same way as their hospital colleagues. Surely they will therefore organise their practices to provide relational continuity? It is, after all, one of the core values of general practice, espoused as such by the Royal College of General Practitioners [5] and the American Association of Family Physicians [6], and until recently general practitioners certainly said that they valued continuity of care [7, 8]. But in England at least, that’s not how they appear to organise their practices. In 2004 UK GPs, who had all been trained in the values of continuity of care, were given a financial incentive to provide prompt appointments. They did so by introducing a system of ‘Advanced Access’ for booking appointments [9] so that prompt appointments became available, but often at the expense of patients being able to see the doctor of their choice. This system of booking has continued in most practices despite the removal of the financial incentive. It is as though the ‘core value’ of relational continuity was only skin deep, and only a small change in administrative incentives led to it being simply forgotten as a priority.

Of course, not all patients have a particular doctor they want to see [10, 11] and doctors may rationalise this change on the basis that many patients are not looking for continuity. Yet among over two million respondents to a recent survey in England, more than half of people in the youngest age group of respondents (18–25) had a particular doctor they preferred to see, rising to over 80% among the elderly [12]. So with clear evidence that our patients want relational continuity, and with continuity being espoused by professional societies as a core value of benefit to patients and doctors alike, how is it so easily shed?

Some of the reasons are easy to see. Doctors are much more likely to work part time than they used to, and in the UK many have professional interests outside clinical care (e.g. teaching, research, healthcare management). So they are simply less available for their patients. Perhaps more insidiously, a generation of young doctors is emerging from hospital training without any experience of having personal responsibility for a defined group of patients. The idea that a problem is one that can always be passed on to someone else is incompatible with an ethos of personal responsibility that is fundamental to relational continuity.

So we have a paradox. Our patients are getting older, more with complex comorbidities that require a personal physician to help coordinate and integrate their care. At the same time, doctors are less able to provide that care. In response to this, health care systems are employing a new generation of healthcare workers whose job appears to be to do what the GP used to do. In the UK, these new workers have titles such as ‘case managers’ or ‘community matrons’. It is in fact impossible for a single person to provide high quality care for people with multiple complex problems, but that does not mean that the family physician should not be at the core of coordinating and integrating a patient’s care. So do doctors value continuity of care? And if so, are they prepared to organise they clinical practice accordingly, albeit within a context where care will also be provided by other members of the team? [13]. Maybe this is a peculiarly English problem. American patients expect to be able to identify ‘my doctor’ and are surprised that English patients may be seen, apparently randomly, by any one of a number of doctors in their general practice.

If I seem to be critical of my colleagues, I also accept blame myself. I have been a GP throughout this period. I reorganised my appointments in 2004 to provide rapid access at the expense of continuity. I contribute to the problem by being a part time academic. I have observed myself believing one thing and doing the opposite. It is exactly this paradox which we need to resolve.

What are the solutions? First, the profession needs to decide whether relational continuity matters. If it doesn’t, let’s drop it from the curriculum. But if it does, then doctors need to take a lead. And there is a range of things that can be done which have been described in detail by Hill and Freeman [14]. These might include:

1. Ensuring that patients understand that doctors can look after them better if they are seeing a patient they know. This is especially important for people from socio-economically deprived populations who have the greatest burden of illness, the greatest need for continuity of care and yet the lowest ability to navigate the administrative barriers which we erect.
2. Change our receptionists’ behaviour and the prompts on our on-line booking systems so that the patient’s ‘own doctor’ becomes the default option.
3. Organise large practices into small teams of two or three doctors who see each others’ patients when one is away. Ensure that patients know about these arrangements. Again, this could be easily introduced into online booking systems which will soon become the booking route of choice for many patients.
4. Allow email contact with doctors so that continuity can be maintained even when the doctor is off site. Many doctors like the idea of email with patients in principle, but are terrified of the workload implications. This is an issue for professional negotiators to resolve: with adequate remuneration, there is no reason why email consultations should not become part of routine care. Indeed, this may increasingly be expected by a younger generation. As my young professional daughter put it: “Why on earth would you want to see a doctor if you could sort it out by email?”
5. Identify patients with particularly complex problems who should only be seen by a restricted number of doctors in a practice. Adjust the appointment system so that they cannot be booked into other doctors. Explain to the patients this means they may have to wait longer for an appointment but they will get better care for their complex problems.
6. Develop better questions on continuity of care in patient surveys and make sure they are included in patient assessments of care. For example, the English GP Patient Survey which samples 2.6 million people annually includes questions on continuity of care and therefore provides a basis for practices benchmarking themselves against other local practices [15].
7. Where countries have arrangements for regular appraisal of doctors or periodic revalidation or recertification, include questions on how a doctor’s practice is organised to provide relational continuity.

I don’t put these forward as a definitive list. But I do suggest that if GPs and their professional leaders believe their own rhetoric around continuity of care, they need to ensure that those values are enacted in daily clinical life.

Finally, what has this to do with integrated care? Everything. Much of the energy around interventions to integrate care seeks to make arrangements that substitute for having a doctor in overall control of the patient’s care. Maybe we should be looking at better ways to provide the personal care that the 1960s TV icon Dr Finlay provided, while still giving patients the benefits of modern medicine.

## From the author

**Martin Roland** is Professor of Health Services Research at the University of Cambridge in the UK. Previously he was Director of the National Primary Care Research and Development Centre and Professor of General Practice at the University of Manchester from 1992 to 2009. His research focuses on developing ways of measuring and improving the quality of primary medical care. Professor Roland has been a practicing GP for over 30 years, currently in a practice in Cambridge. In 2002 and 2003, he was an advisor to the Department of Health and the British Medical Association on the development of the Quality and Outcomes Framework, a major pay for performance scheme for UK GPs.
